# Prognostic value of FDG-PET indices for the assessment of histological response to neoadjuvant chemotherapy and outcome in pediatric patients with Ewing sarcoma and osteosarcoma

**DOI:** 10.1371/journal.pone.0183841

**Published:** 2017-08-25

**Authors:** Clement Bailly, Rodolphe Leforestier, Loic Campion, Estelle Thebaud, Anne Moreau, Francoise Kraeber-Bodere, Thomas Carlier, Caroline Bodet-Milin

**Affiliations:** 1 Nuclear Medicine Department, University Hospital, Nantes, France; 2 Nantes-Angers Cancer Research Center CRCNA, University of Nantes, INSERM UMR892, CNRS-UMR6299, Nantes, France; 3 Biometrics Department, ICO René Gauducheau Cancer Center, Saint Herblain, France; 4 Pediatric Oncology Department, University Hospital, Nantes, France; 5 Pathology Department, University Hospital, Nantes, France; BIDMC, UNITED STATES

## Abstract

**Purpose:**

The objective of this retrospective work was to evaluate the prognostic value on histological response and survival of quantitative indices derived from FDG-PET performed before and after chemotherapy (CHT), in a homogeneous pediatric Ewing sarcoma (EWS) and Osteosarcoma (OST) population.

**Methods:**

Thirty-one patients with EWS and 31 with OST were included. All patients were treated with neoadjuvant CHT, and underwent surgery for local control. All patients had FDG-PET at diagnosis and after CHT, prior to surgery. Several parameters were evaluated: SUVmax, SUVpeak, SUVmean, metabolic tumor volume, total lesion glycolysis, 7 textural features and 3 shape features (SF). The segmentation was performed using an adaptive approach. Results were compared to histopathological regression of the resected tumor and to clinical follow-up for survival evaluation.

**Results:**

For EWS, univariate analysis did not highlight any prognostic value on histological response, or survival regardless of all the considered metrics. For OST, only one of the SF, namely elongation, was significantly associated with PFS and OS on both univariate and multivariate analysis (PFS: p = 0.019, HR = 5.583; OS: p = 0.0062, HR = 7.113).

**Conclusion:**

Only elongation determined on initial FDG-PET has a potential interest as a prognostic factor of PFS and OS in pediatric OST patients. Unlike recent studies of the literature realized in adult population, all the metrics reveal limited additional prognostic value in pediatric EWS patients. This seems to reinforce the question of whether children experience different subtypes of the same pathologies than older patients, with different outcomes.

## Introduction

Ewing sarcoma (EWS) and osteosarcoma (OST) are the most common pediatric bone sarcomas[1,2]. Over the last three decades, the survival outcome of these patients has improved significantly, due to the introduction of aggressive preoperative multiagent chemotherapy (CHT)[3,4]. However, the development of new risk-adapted strategies to select worse prognosis patients who might benefit from more intensive therapy, still appears essential[5]. Except for the presence of metastatic disease at time of diagnosis and for the histopathologic tumor necrosis after CHT which constitute important prognostic factors on both progression-free survival (PFS) and overall survival (OS), the other proposed prognostic factors varied substantially in different series[6,7]. Within this context, imaging modalities and more particularly positron emission tomography using 18-fluoro-deoxy-glucose (FDG-PET) might represent valuable tools. Some studies were focused on FDG-PET performance for accurate staging of the extent of disease and patient’s response to neoadjuvant CHT[8–11], however only a few have assessed the usefulness of metabolic and volumetric information as prognostic factors[12–16]. Moreover, most of them explored heterogeneous populations mixing different histological subtypes, CHT regimens or age groups. The objective of this retrospective work was thus to evaluate the prognostic value of FDG-PET at baseline and after CHT induction in a pediatric bone sarcoma population including homogeneous subgroups of EWS and OST.

## Materials and methods

### Patients

All children, treated for histological proven EWS and OST, at University Hospital of Nantes between 2004 and 2014 were included. All children were treated according to international risk-adapted protocols with homogeneous CHT in each subgroup (OS 2005 and OS 2006 for OST patients; EUROEWING 99 for EWS patients). For ESW, the neoadjuvant chemotherapy regimen included 6 courses of VIDE (Vincristine, Ifosfamide, Doxorubicin, Etoposide) according to Euro-EWING 99 protocol.For OST, the neoadjuvant chemotherapy regimen included 6 courses of Methotrexate and 2 courses of VI (VP16, Ifosfamide) according to the OS 2006 protocol.

Initial staging and response assessment were performed according to these protocols. Whole-body FDG-PET were additionally realized prior to therapy initiation and after induction CHT, prior to surgery, to assess therapeutic response but not used in decision-making strategy.In cases of suspected metastases on initial FDG-PET not seen on initial conventional imaging (CI), contrast-enhanced CT or MRI were performed for assessment of the lesions.

### Conventional imaging modalities

CI consisted, in addition to clinical examination and bone marrow biopsy, of chest X-ray, contrast enhanced CT, contrast enhanced MRI of the primary tumor site and additional regions when clinically indicated and bone scintigraphy (BS) after intravenous injection of 10 MBq(270μCi)/kg of 99mTc—labeled phosphonates (Symbia T or E-cam, Siemens). CI were obtained at baseline, and after induction CHT. CI response was determined according to RECIST 1.1 criteria[17] on CT and using decreasing extent of marrow invasion, reduction of tumor volume and decreasing in the amount of associated oedema on MRI to classifypatients in 4 response groups: complete response (CR), partial response (PR), stable disease (SD) and progressive disease (PD).

### FDG-PET imaging

Children fasted at least 4 hours before 18F-FDG injection and blood glucose was controlled prior to the injection. Images’ acquisition was performed, on a Discovery LS PET/CT (GE Medical Systems) 60 to 80 min after intravenous injection of 5–7 MBq(135–189μCi) / kg of 18F-FDG or on a Biograph mCT(Siemens) after intravenous injection of 3 MBq(81μCi)/kg of 18F-FDG.

All FDG-PET images were retrospectively reviewed on a dedicated workstation (PLANET^®^Onco-Solution, Dosisoft, France) and evaluated in consensus by two experienced readers.

Positive FDG-PET was defined as abnormal uptake greater than surrounding background not explained by normal organ uptake.

Different quantitative metrics were extracted on initial FDG-PET and before surgery FDG-PET, measured with a volume of interest (VOI) covering the entire tumor as visualized by increased FDG uptake. If the tumor was not delineable in the follow-up examination, the VOI of the baseline scan was reproduced using anatomical landmarks.

The following metrics were extracted:

- SUVmax, defined as theStandard-Uptake-Value(SUV) value of the maximum intensity voxel within the VOI.
- SUVmean, defined as the average measure of SUV within calculated boundaries of a tumor,
- SUVpeak, defined as the average SUV within a fixed 1-cm^3^ spherical VOI centered on the highest-uptake part of the tumor,
- Metabolic Tumor Volume (MTV) defined as the functional volume determined on functional imaging,
- Total Lesion Glycolysis (TLG) defined as the product of SUVmean and MTV,
- Heterogeneity quantification through 6 textural features (TF). The chosen TFs are among the most widely used in recent publications. They were also chosen because of a proven robustness against different acquisition/reconstruction settings[18,19].A 64-grey-levels quantization was used for resampling purpose.A list of all the TFs studied is provided in Table 1.
- Shape features (SF) were calculated directly from the segmented VOI. SF provided information on the regularity and complexity of an object by quantifying its self-similarity level[20].

**Table 1:** List of heterogeneity and shape indices.

Matrix name or class	Featurename	Description
**Grey level co-occurence matrix (GLCM)**	Homogeneity	Local homogeneity of paired voxels
	Entropy	Local measure of information content
	Dissimilarity	Linearweightedcontrast
**Grey level run length matrix (GLRLM)**	High Grey Level Run Emphasis (HGRE)	Distribution of high grey-level run
**Grey level size zone matrix (GLSZM)**	Zone Length Non Uniformity (ZLNU)	Measure of size zone variability
	Short-Zone High Gray-level Emphasis (SZHGE)	Distribution of small zone of high grey-level
**Shape features**	Elongation	Indication of the flatness of the shape
	Sphericity	Measure of the spherical shape (roundness)
	Compactness	Measure of the similarity between shape of interest and a perfect ellipse

The GLCM and GLRLM were calculated from 13 directions with one-voxel displacement. The final textural features were computed by taking into account all 13 directions simultaneously. A 64-grey-levels quantization was used for resampling purpose.

For each metric requiring a segmentation step, the adaptive approach proposed by Vauclin et al was chosen[21].

For each metric, the baseline value (i.e. for SUVmax: SUVmax_1_), the post-CHT value (i.e. for SUVmax: SUVmax_2_) and the reduction between both exams (i.e. for SUVmax: ΔSUVmax) were considered.

Metabolic response assessment was also assessed according to PERCIST criteria[22]. SUVpeak normalized on body weight was used rather than SULpeak calculated by normalization for the lean-body-mass as there is yet no agreement on the way by which this index should be determined.

### Histologic and clinical analysis

Histological regression of the resected primary tumors after neoadjuvant CHT was evaluated in the resected specimen by 2 experienced pathologists, according to Salzer-Kuntschik score[23]. Patients with <10% viable tumor cells (Salzer-Kuntschik I–III) were defined as responders whereas patients with ≥10% viable tumor cells were regarded as non-responders (Salzer-Kuntschik IV–VI).

Classic prognostic factors such as primary tumor site (axial/pelvic vs peripheral), histologic subtype for OST, presence of metastatic disease at diagnosis were also evaluated for their impact on OS and PFS.

### Statistics

At initial staging, CI and FDG-PET results were compared to the status of the disease determined by histopathologic examination of lesions (if available) or clinical and imaging follow-up allowing determination of sensitivity and specificity.

For post-CHT exams, results were compared to histopathological regression of the resected tumor as defined by Salzer-Kuntschik using Spearman analysis.

End points studied were PFS and OS, determined by clinical and imaging follow-up. PFS was defined as the number of days after the initiation of CHT until disease recurrence or death from any cause. OS was defined as the number of days from the initiation of CHT until death. Patients who were alive and in whom disease did not recur were censored at the time of their last documented clinical communication.

The Cox proportional hazard regression model was used to estimate the hazard ratio (HR) in univariate and multivariate analyses.

Only p values ≤ 0.05 were considered as statistically significant.

### Ethical approval

Written and informed consent was obtained from each patient and parents. Local ethics committee of Nantes University Hospital (France) approved this study.

## Results

### Patient population

Patients’characteristics are presented in Table 2. Sixty-two children with histologically-proven EWS or OST were included in this study. Median age at the time of diagnosis was 13.9 years (range 8–17 years). All patients had FDG-PET at diagnosis and were treated with CHT. Eleven patients (35% of subgroup) with EWS and twelve (38% of subgroup) with OST presented with detectable distant metastases.

**Table 2:** Patients’ characteristics.

Parameter		All patients	EWS	OST
No. of patients		62	31	31
Gender	Male	31 (50%)	18 (58%)	13 (42%)
	Female	31 (50%)	13 (42%)	18 (58%)
Age	Median	13.9	14.7	12.8
	SD	+/-3	+/- 3.2	+/- 2.9
Metastasis at diagnosis	Bone lesions	9 (14%)	5 (16%)	4 (13%)
	Lung lesions	14 (23%)	6 (19%)	8 (26%)
	Skip metastasis	4 (6%)	2 (6%)	2 (6%)
	Others	1 (2%)	0	1 (2%)
Primary tumor site	Axial/pelvic	13 (21%)	13 (42%)	0
	Peripheral	49 (79%)	18 (58%)	31 (100%)

EWS: Ewing sarcoma; OST: Osteosarcoma; SD: Standard Deviation

Histologic subtypes of the OST tumors were osteoblastic (n = 23), chondroblastic (n = 7) and telangiectatic (n = 1).

Forty-three (70%) children had FDG-PET after induction CHT (24 patients with OST and 19 patients with EWS). Only these latter were included in the post induction FDG-PET analysis and its correlation with histological response. All patients with FDG-PET after induction therapy underwent surgical resection. Fifty-six (90%) of all children underwent surgery for local control. Twelve children (19%; 7 OST and 5 EWS) showed a poor histological response (Salzer-Kuntschik IV–VI)within the resected primary tumors and were considered as non-responders after induction chemotherapy. Median follow up was 5 years. Of the 62 patients, 21 children (33%; 9 EWS, 12 OST) relapsed and 12 (19%; 5 EWS, 7 OST) died.

### Initial staging

#### FDG-PET and CI at initial staging

FDG-PET and CI were equally effective in the detection of primary tumors (accuracy, 100%). FDG-PET was superior to CI for detection of bone metastases in both EWS (sensitivity, 80% vs. 60%) and OST (sensitivity, 100 vs. 20%), whereas CI was more reliable than FDG-PET in depicting lung metastases (sensitivity, 100% v 50%, respectively).

#### Semi-quantitative metrics derived from initial FDG-PET

Results for semi-quantitative metrics derived from initial FDG-PET are presented in Table 3.

**Table 3:** Semi-quantitative metrics derived from initial FDG-PET.

	EWS			OST		
	Average	Min	Max	Average	Min	Max
SUVmax_1_	7.7	1.6	18.4	12.7	4.9	35.3
SUVpeak_1_	5.7	1.3	14.2	9.5	3.6	26.4
SUVmean_1_	3.8	0.9	9.5	5.4	2.5	13.8
TLG_1_	527.2	7.5	3249.7	570.7	60.9	2089.4
MTV_1_	115.2	3.6	469.6	104.0	10.4	262.2
Homogeneity	0.211	0.127	0.311	0.224	0.137	0.390
Entropy	6.188	3.865	7.314	6.564	5.12	7.212
Dissimilarity	9.438	4.327	17.765	8.424	3.546	14.898
HGRE	483.488	278.577	834.277	368.766	84.055	763.521
ZLNU	810.604	23.256	3389	698.094	145.73	1971.981
SZHGE	416.971	239.241	751.213	319.019	121.414	693.061
Sphericity	0.439	0.284	0.557	0.403	0.217	0.62
Elongation	1.489	1.072	3.302	1.561	1.016	2.884
Compactness	0.091	0.023	0.173	0.079	0.01	0.239

EWS: Ewing sarcoma; OST: Osteosarcoma; SUV: Standard Uptake Value; TLG: Total Lesion Glycolysis MTV: Metabolic tumor volume; HGRE: High Grey Level Run Emphasis; SZHGE: Short-Zone High Gray-level Emphasis; ZNLU: Zone Length Non Uniformity

#### Prognostic value on PFS and OS

For EWS, only presence of bone and lung metastasis revealed prognostic value on PFS on univariate analysis (Table 4) confirmed on multivariate analysis (Table 5). None of all the considered metrics derived from initial FDG-PET showed any other prognostic value on PFS or OS.

**Table 4:** Prognostic values (*p value* and *Hazard Ratios* when p-value < 0.05) of classical prognostic factors derived from initial staging and of metrics derived from initial FDG-PET, on univariate analysis.

	EWS				OST			
Parameter	PFS		OS		PFS		OS	
	*p-value*	*Hazard Ratio*	*p-value*	*Hazard Ratio*	*p-value*	*Hazard Ratio*	*p-value*	*Hazard Ratio*
Bone Metastasis	**0.0008**	11.466	0.053	6.074				
Lung Metastasis	**0.0143**	5.941	0.619	-	**0.003**	6.427	**0.01**	6.26
Histologicsubtypes	-	-	-	-	**0.018**	0.265	**0.04**	0.247
Elongation					**0.0044**	5.684	**0.0062**	7.113

Significant p-value in bold.

For EWS, anatomic primary site corresponds to primary tumor site (axial/pelvic vs peripheral).

For OST, histologic subtypes compares Osteoblastic vs Chondroblastic/Telangiectatic subtypes.

EWS: Ewing sarcoma; OST: Osteosarcoma

**Table 5:** Prognostic values (*p value* and *Hazard Ratios* when significant p-value) of classical prognostic factors derived from initial staging and of metrics derived from initial FDG-PET, on multivariate analysis.

	EWS				OST			
Parameter	PFS		OS		PFS		OS	
	*p-value*	*Hazard Ratio*	*p-value*	*Hazard Ratio*	*p-value*	*Hazard Ratio*	*p-value*	*Hazard Ratio*
Bone Metastasis	**0.0002**	29.698	NRM	-	-	-	-	-
Lung Metastasis	**0.0016**	18.78	-	-	**0.031**	5.308	NRM	-
Histologicsubtypes	-	-	-	-	**0.041**	0.301	NRM	-
Elongation	-	-	NRM	-	**0.019**	5.583	**0.0062**	7.113

Only significant or nearly significant indices on univariate analysis were included on the multivariate analysis. Significant p-value in bold.

For OST, histologic subtypes compares Osteoblastic vs Chondroblastic/Telangiectatic subtypes.

EWS: Ewing sarcoma; OST: Osteosarcoma; NRM: not retained in model.

Only factors with significant p-value are presented in Table 4. P-value calculated for all factors are presented in S1 Table.

For OST, univariate analysis showed that presence of lung metastasis, histologic subtypes and one of the SF, namely elongation, were significantly associated with both PFS and OS. Multivariate analysis confirmed that the presence of lung metastasis, histologic subtype and elongation were independent factors for PFS whereas elongation was the only significant factor for OS(PFS: p = 0.019, HR = 5.583; OS: p = 0.0062,HR = 7.113) (Tables 4 and 5).

**Fig 1:**
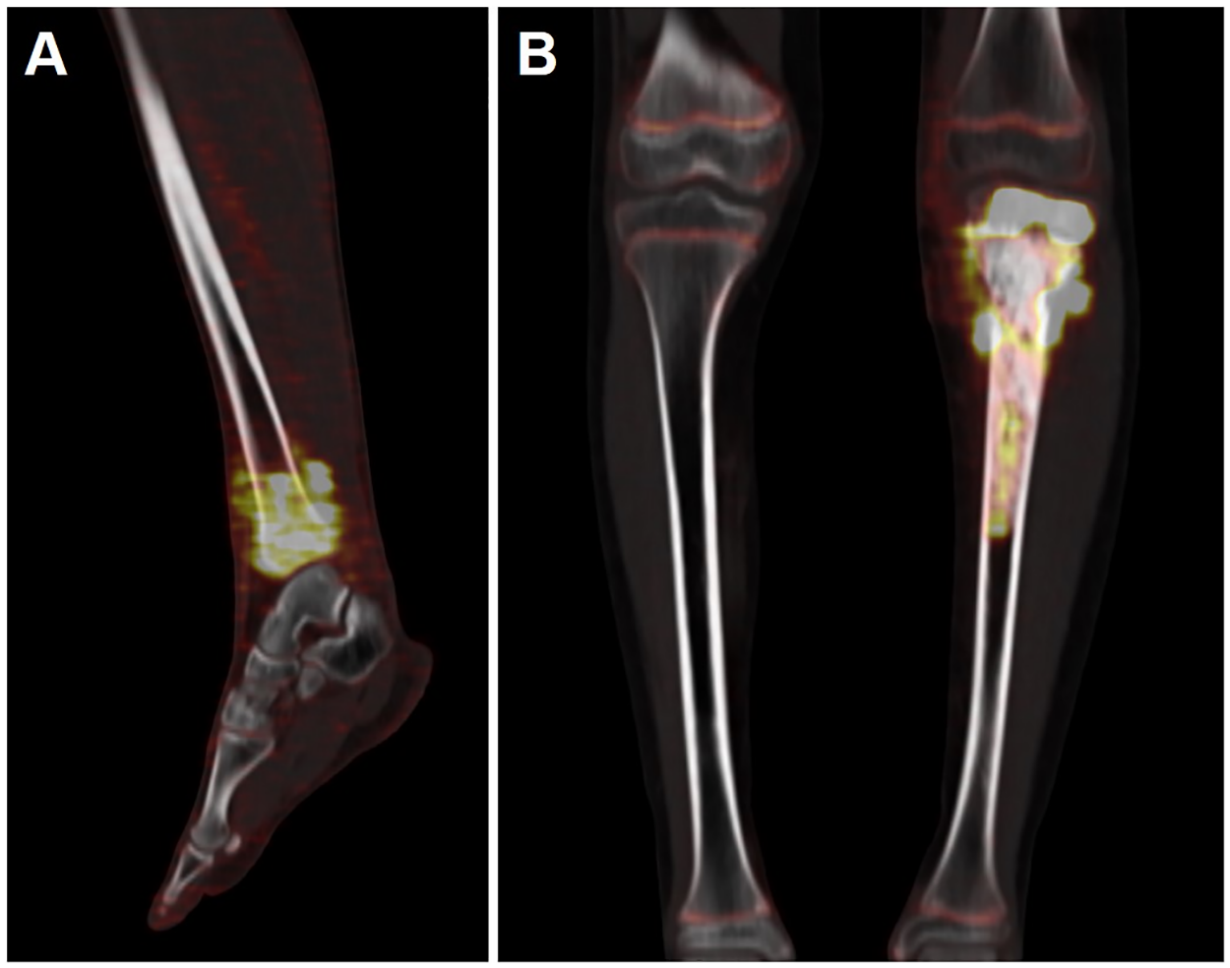
Shape elongation’s measure. Shape elongation’s measure is based on the ratio of the longer and shorter edges of the minimum area bounding rectangle for the measured shape. An elongation factor of 1 represents an ideal case with maximum symmetry. Thus, in example (A), in a patient with Ewing Sarcoma of the left tibia, the elongation factor measured on the initial FDG-PET scan was 1.32. Greater values express greater elongation of the ROI volume such as in example (B) in a patient with osteosarcoma of the left tibia with an elongation factor of 2.68.

Two examples of shape elongation’s measure are presented in Fig 1.

### Post CHT evaluation

#### FDG-PET and CI after CHT

After induction CHT, FDG-PET found 32% CR, 63% PR and 5% SD according to PERCIST criteria in EWS whereas CI found 25% CR, 29% PR and 46% SD. In OST, FDG-PET found 0% CR, 87% PR and 13% SD according to PERCIST criteria whereas CI found 0% CR, 30% PR and 70% SD.

#### Semi-quantitative metrics derived from post-induction CHT FDG-PET

Results for semi-quantitative metrics derived from post-induction FDG-PET are presented in Table 6.

**Table 6:** Semi-quantitative metrics derived from post-CHT FDG-PET.

	EWS			OST		
	Average	Min	Max	Average	Min	Max
SUVmax_2_	2.8	0.7	6.8	3.6	1.8	8.0
ΔSUVmax	-60.3	-82.4	10.6	-67.1	-93.1	-23.8
SUVpeak_2_	1.9	0.5	5.1	2.8	1.4	6.1
ΔSUVpeak	-63.8	-86.6	-15.8	-64.8	-92.7	-14.4
SUVmean_2_	1.4	0.0	4.7	1.6	0.9	3.8
ΔSUVmean	-64.3	-100.0	20.0	-65.5	-91.7	-20.8
TLG_2_	32.3	0.0	196.2	267.9	54.8	686.8
ΔTLG	-89.3	-100.0	9.9	-29.0	-88.8	395.4
MTV_2_	24.5	0.0	146.4	181.4	41.8	472.5
ΔMTV	-66.4	-100.0	233.9	157.4	-36.5	2519.1

EWS: Ewing sarcoma; OST: Osteosarcoma; SUV: Standard Uptake Value; TLG: Total Lesion Glycolysis MTV: Metabolic tumor volume

#### Prognostic value on histologic response, PFS and OS

After CHT, neither absolute metrics and reductions, nor PERCIST criteria and CI response were significantly associated with histologic response or survival for either EWS or OST (S2 and S3 Tables).

**Fig 2:**
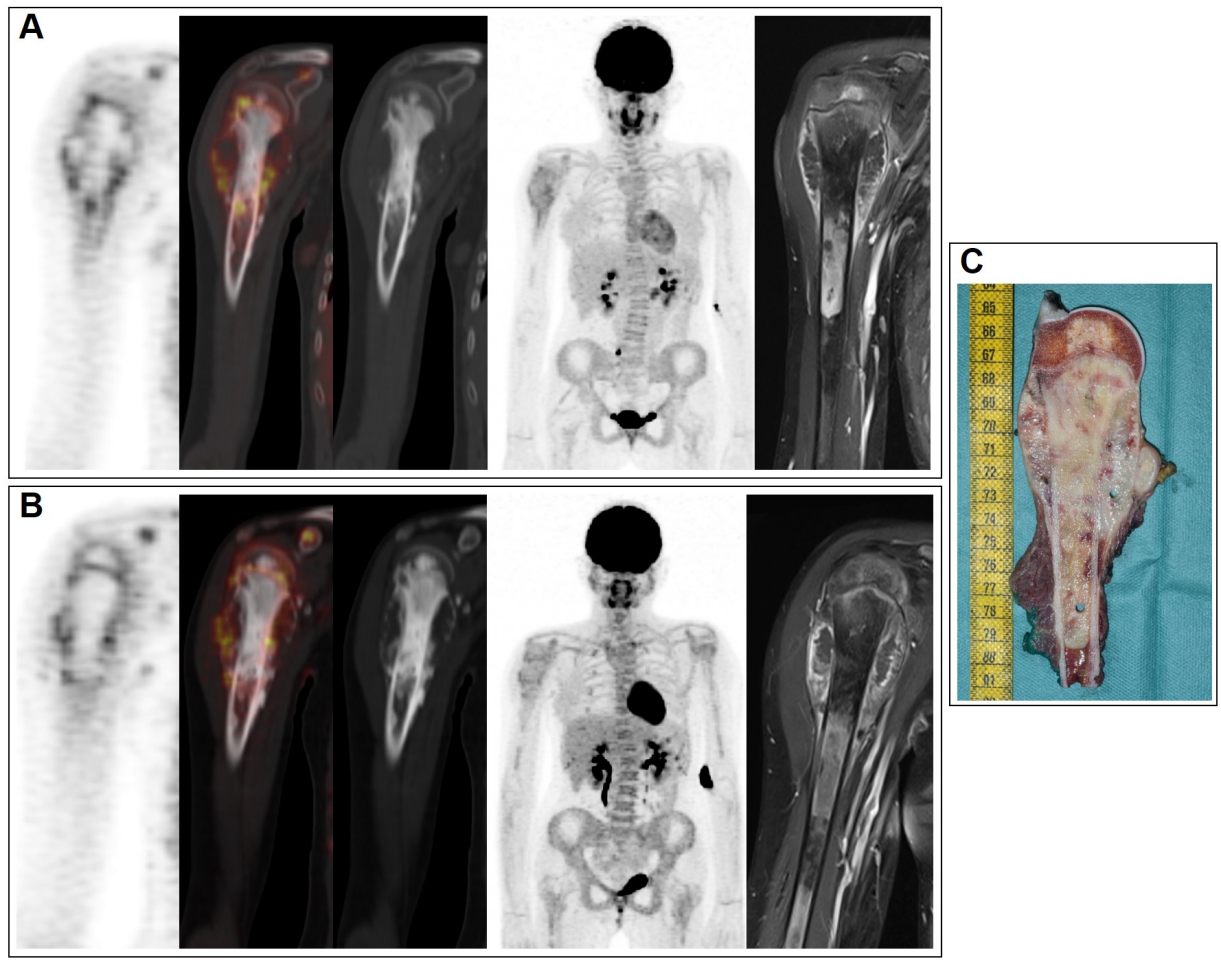
Various explorations conducted in a patient with osteosarcoma of the right upper arm. A 14-year-old girl with osteosarcoma of the right upper arm visualized by FDG-PET scans acquired before chemotherapy (A) and after chemotherapy (B) (left column, coronal view of PET; second column, fused PET/CT; third column, CT images; fourth column, the maximum intensity projections of PET; right column, local MRI unenhanced T1- weighted). After neoadjuvant chemotherapy (B), local MRI showed a stable tumor size and FDG-PET remained similar to baseline. Despite this stable disease on imaging, the removed tumor specimen (C) was almost completely composed of necrotic tissue (96%).

**Fig 3:**
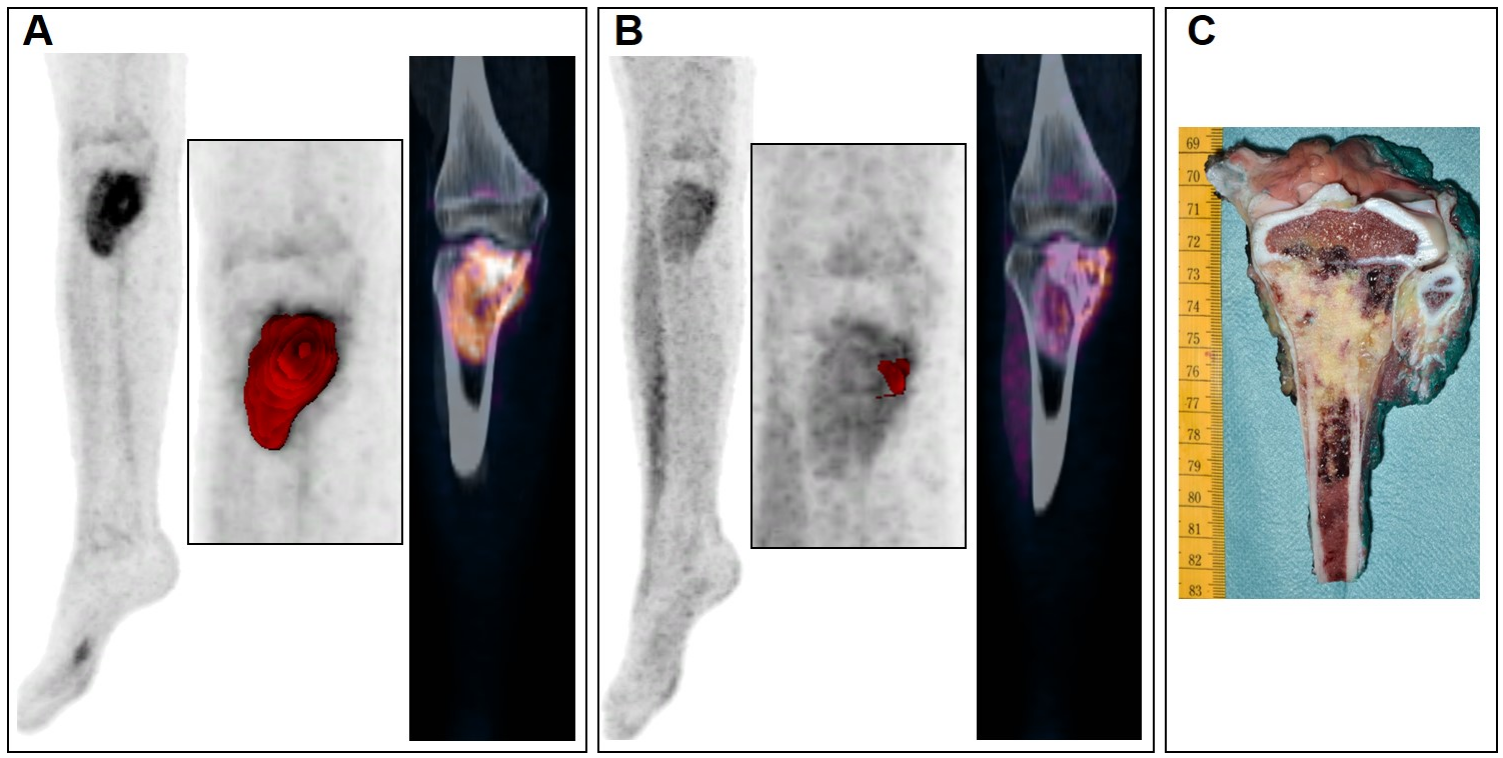
Various explorations conducted in a patient with osteosarcoma of the right tibia. FDG-PET scans acquired before (A) and after chemotherapy (B) in a patient with osteosarcoma of the right tibia. The maximum intensity projections of PET (first columns) and the coronal views of fused PET/CT images (third columns) showed evident significant reduction in MTV (second columns) using an adaptative threshold, after chemotherapy. The removed tumor specimen (C) was almost completely composed of necrotic tissue (99%).

Examples of various explorations conducted in two patients with osteosarcoma are presented in Figs 2 and 3.

## Discussion

Although there are conflicting data regarding prognostic factors in OST and EWS, their identification to define different risk groups remains very important. A number of clinical and pathologic features with prognostic significance have been reported including age, gender, tumor site, size, stage, histologic subtype and histologic response to pre-operative CHT[6,7]but often with contradictory results due to the lack of uniformity in patient analyses and methods. In particular, despite a potential impact of age on the prognosis of bone sarcoma patients, some of these studies mixed adult and pediatric populations[10,16,24]. In this context, the purpose of this retrospective work was thus to evaluate the prognostic value of FDG-PET in a pediatric bone sarcoma population including homogeneous subgroups of EWS and OST both for staging and response assessment.

According to the currentprotocols, MRI, CT, BS and radiography represented the main imaging modalities performed in pediatric bone sarcomas’staging[25]. FDG-PET’s exact role in the management of these patients remains unclear. Several studies revealed that it could improve the accuracy of bone cancer staging with a high sensitivity for detecting skeleton and soft tissue lesions[10,11]. In our study, FDG-PET was clearly superior in detecting bone lesions (sensitivity, 90% v 40% for CI) and prominently in the subgroup of OST patients (sensitivity, 100% v 20% for CI). Whole body acquisitionpartly explained theFDG-PET’s good performances by depicting distant lesions not visualized on centered morphologic exams. CI and particularly CT were superior than FDG-PET in detecting small pulmonary metastases, because of known technical limitations such as respiratory motion artifacts and partial volume effect (sensitivity, 100% for CI vs 50% for FDG-PET). CI and particularly MRI were clearly superior in detecting skip metastasis (sensitivity, 100% for CI vs 50% for FDG-PET). FDG-PET and CI were equally effective in the detection of primary tumors (accuracy, 100%). Our results thus confirmed the complementary character of CI and FDG-PET in bone sarcomas’ initial staging but also the need of FDG-PET’s realization at diagnosis with a probable significant impact in terms of prognosis, given the importance of an optimal initial treatment.

It has been shown that primary tumor’s FDG uptake measured by SUVmax at staging correlates with the grade of tumor differentiation [12,15,16,26]. These studies also reported prognostic discrimination, the cellular composition of sarcomas being frequently heterogeneous with the most aggressive sarcoma cells determining the outcome of the patient. Yet, all of these works were realized in adult population or mixed adult and pediatric populations: for example, Franzius et al retrospectively studied a population with a median age of 14 years-old (range: 5–41 years-old)[26]. In our study, the SUV measures at baseline do not seem to be a prognostic factor for both EWS and OST pediatric patients. The prognosis impact of FDG uptake might be different in adult or children patients as none of the previous studies were realized in a strictly pediatric population. In the literature, even if for some, the prognostic relevance of patient age remains controversial[27], older age seemsassociated with a worse outcome in both OST[28,29] and EWS[30]. Moreover, patients over the age of 16 years appear to statistically present larger tumors or a high frequency of pelvic primary tumor sites or of metastatic disease [30,31].The Children’s Oncology Group explored a group of 1054 OST patients, of whom 128 were aged 18–30 years at diagnosis[28]. Significantly more relapse and poorer PFS or OS were observed in this subgroup,not explained in this study by tumor location, metastatic disease or histologic response. The authors concluded that unfavorable tumor biology in 18 to 30 years old should be further investigated. Actually, one could hypothesize that the discrepancies of SUVs’ prognostic impact between children and adults populations, in the literature and our study, might reflect thesedifferences in tumor biology.

Several previous studies have also assessed the prognostic value of tumor size or tumor volume of OST and EWS at diagnosis using different imaging modalities. A few studies focused on initial metabolic volume assessed on FDG-PET in OST patients and reported the prognostic value of TLG and MTV using different cutoff values in line with data measured by MR imaging[12,13]. In our study, baseline TLG and MTV of the primary tumor in both subgroups didn’t predicted survival. These heterogeneous results of the literature could be explained by the low number of studies exploring the prognostic value of volume-based metrics but also by the different methods used as none is actually considered as the reference[32].

Besides, many studies have, recently, focused on the heterogeneity of the radiotracer’s distribution within the tumor volume using FDG-PET images[32] and investigated the use of TF at diagnosis in a number of solid cancers[32]. In human sarcomas, a measure of tumor shape information was also explored and appeared to provide an independent prognostic indicator of duration of PFS and OS[20]. In our study, one of the measured SF, namely elongation (Fig 1), was significantly associated with PFS and OS in univariate analysis for OST, as well as lung metastases and histologic subtypes, known prognostic factors in this pathology. Multivariate analysis confirmed that these factors were independent factors for PFS whereas only elongation was a significant factor for OS, confirming its potential predictive interest. For EWS, only previously described factors (presence of bone and lung metastases) revealed prognostic value on PFS on multivariate analysis.

Many authors investigated the role of different imaging modalities in the assessment of response to preoperative CHT in patients with primary bone sarcomas and particularly their ability to reflect the degree of tumour necrosis on resected specimens. As previously stated, the presence of <10% viable cells is one of the main prognostic factor in both OST and EWS. In papers comparing FDG-PET to CI performances, because OST and EWS lesions frequently do not change in size in response to CHT,the decrease in glycolytic activity generally seemed better correlated with histological necrosis, rather than the reduction in lesion size as assessed by post-CHT CI[9]. Yet, heterogeneous results were reported regarding the metrics used. Most authors showed an association between histologic good response and SUVmax_2_≤2–2.5 or ΔSUVmax[16,24], while, in our study and in Gaston et al’s work, neither SUVmax_2_ nor ΔSUVmax were significantly associated with histologic response for ESW or OST[33]. Two explanations could be raised. Firstly, as previously described, these studies were mainly focused on adult or mixed populations for which SUVmax at diagnosis had a prognostic value. Secondly, high FDG uptake after therapy may be either ascribed to persistence of viable tumoral cells or to the presence of inflammation and reactive fibrosis frequently observed in sites of healing normal bone after successful treatment[34]. These findings are also concordant with those obtained with other FDG uptake measurement tools or SUV-based interpretation criteria which showed a great number of discrepancies in the evaluation of response.One example is shown in Fig 2, in a patient with osteosarcoma, in whom,despite stable disease on imaging, the removed tumor specimen was almost completely composed of necrotic tissue.

Other potential metrics have also been explored to assess therapeutic response in solid tumors such as volume-based PET parameters.ΔMTV of OST or EWS lesions seemed to show a good correlation with histological response [12,14,33]. Yet, in our study, the MTV or TLG reduction were not able to significantly discriminate histopathological responders in both subgroups, unlike the example shown in Fig 3. As previously discussed, the absence of sufficiently robust delineation techniques for tumor volume segmentation makes consensus development difficult. Moreover, as previously reported[33], EWS patients usually exhibited a greater reduction in lesion volume after CHT, as compared to OST. This can probably explained by the larger soft tissue component associated with EWS, compared to OST which have a large extracellular matrix of bone and osteoid requiring active resorption.

Nevertheless, the present study had some inherent limitations. It was a single-center retrospective study with a relatively small number of patients and thus a small number of events as both OST and EWS are relatively uncommon malignancies, which makes accumulation of cases for a cohort difficult. Moreover, two different PET systems were used for FDG-PET acquisitions. Yet, the uniformity of pediatric patients with newly diagnosed malignancy, the homogeneity of treatments and biopsy proof of all primary diseases constituted real strengths of our study.

In conclusion, firstly, our results confirmed the usefulness of FDG-PET in pediatric bone sarcomas’ initial staging with a likely significant impact in terms of prognosis, given the prognostic value of bone or lung metastases in EWS and OST and the importance of an optimal initial staging. Secondly, unlike recent studies of the literature realized in adult population, we demonstrated that all the “usual” metrics revealed limited additional prognostic value in pediatric EWS and OST patients reinforcing the suggestion that whether children and adolescents experience different subtypes of the same pathologies than older patients, with different outcomes.This also underlines the need to take age into consideration together with other well-known prognostic factors in a treatment algorithm based on FDG-PET data such as the one proposed recently by Palmerini et al[15]. Finaly, our study revealed the potential interest of a SF called elongation determined on initial FDG-PET as a prognostic factor of PFS and OS in pediatric OST patients, which again indicates that extracting more advanced image features from FDG-PET, provides complementary and additional value and reinforces the potential role of FDG-PET in the development of new risk-adapted strategies.

## Supporting information

S1 Table Prognostic values (p value and Hazard Ratios when p-value < 0.05) of classical prognostic factors derived from initial staging and of metrics derived from initial FDG-PET, on univariate analysis. Significant p-value in bold. For EWS, anatomic primary site corresponds to primary tumor site (axial/pelvic vs peripheral).For OST, histologic subtypes compares Osteoblastic vs Chondroblastic/Telangiectatic subtypes.(EWS: Ewing sarcoma; OST: Osteosarcoma; SUV: Standard Uptake Value; TLG: Total Lesion Glycolysis MTV: Metabolic tumor volume; HGRE: High Grey Level Run Emphasis; SZHGE: Short-Zone High Gray-level Emphasis; ZNLU: Zone Length Non Uniformity).
(DOC)

S2 Table Prognostic values (p value) of indices derived from post-CHT FDG-PET on univariate analysis. No Hazard Ratio is shown as no significant p-value was observed.(EWS: Ewing sarcoma; OST: Osteosarcoma; CI: conventional imaging; SUV: Standard Uptake Value; TLG: Total lesion glycolysis; MTV: Metabolic tumor volume).
(DOC)

S3 Table Correlations (Spearman coefficients) between delta and histological regression. (EWS: Ewing sarcoma; OST: Osteosarcoma; SUV: Standard Uptake Value; TLG: Total Lesion Glycolysis MTV: Metabolic tumor volume).
(DOC)
