# Supplementary material for: Prognostic value of FDG-PET indices for the assessment of histological response to neoadjuvant chemotherapy and outcome in pediatric patients with Ewing sarcoma and osteosarcoma
Source: PLoS One. 2017 Aug 25;12(8):e0183841. doi: 10.1371/journal.pone.0183841 (PMC5571925; doi:10.1371/journal.pone.0183841)
Supplement: S1 Table — Significant p-value in bold. For EWS, anatomic primary site corresponds to primary tumor site (axial/pelvic vs peripheral).For OST, histologic subtypes compares Osteoblastic vs Chondroblastic/Telangiectatic subtypes.(EWS: Ewing sarcoma; OST: Osteosarcoma; SUV: Standard Uptake Value; TLG: Total Lesion Glycolysis MTV: Metabolic tumor volume; HGRE: High Grey Level Run Emphasis; SZHGE: Short-Zone High Gray-level Emphasis; ZNLU: Zone Length Non Uniformity). (DOC) [file pone.0183841.s001.doc]

|  | **EWS** | | | | **OST** | | | |
| --- | --- | --- | --- | --- | --- | --- | --- | --- |
| Parameter | PFS | | OS | | PFS | | OS | |
|  | *p-value* | *Hazard Ratio* | *p-value* | *Hazard Ratio* | *p-value* | *Hazard Ratio* | *p-value* | *Hazard Ratio* |
| Anatomic primary site | 0.5808 | - | 0.9528 | - | - | - | - | - |
| Bone Metastasis | **0.0008** | 11.466 | 0.053 | 6.074 | 0.342 | - | 0.164 | - |
| Lung Metastasis | **0.0143** | 5.941 | 0.619 | - | **0.003** | 6.427 | **0.01** | 6.26 |
| Skip Metastasis | 0.5202 | - | 0.964 | - | 0.341 | - | 0.106 | - |
| Histologic subtypes | - | - | - | - | **0.018** | 0.265 | **0.04** | 0.247 |
| SUVmax_1_ | 0.398 | - | 0.32 | - | 0.399 | - | 0.877 | - |
| SUVpeak_1_ | 0.617 | - | 0.349 | - | 0.564 | - | 0.982 | - |
| SUVmean_1_ | 0.204 | - | 0.913 | - | 0.354 | - | 0.879 | - |
| TLG_1_ | 0.98 | - | 0.933 | - | 0.964 | - | 0.415 | - |
| MTV_1_ | 0.533 | - | 0.976 | - | 0.422 | - | 0.153 | - |
| Homogeneity | 0.7407 | - | 0.996 | - | 0.5561 | - | 0.6432 | - |
| Entropy | 0.984 | - | 0.9972 | - | 0.6298 | - | 0.696 | - |
| Dissimilarity | 0.8179 | - | 0.936 | - | 0.5493 | - | 0.7061 | - |
| HGRE | 0.5248 | - | 0.8135 | - | 0.7728 | - | 0.5103 | - |
| SZHGE | 0.5662 | - | 0.9965 | - | 0.687 | - | 0.5971 | - |
| ZLNU | 0.581 | - | 0.7549 | - | 0.9111 | - | 0.6474 | - |
| Elongation | 0.0504 | - | 0.0782 | - | **0.0044** | 5.684 | **0.0062** | 7.113 |
| Sphericity | 0.258 | - | 0.6791 | - | 0.2344 | - | 0.1262 | - |
| Compactness | 0.1628 | - | 0.4945 | - | 0.285 | - | 0.2865 | - |

**S1 Table**: **Prognostic values (*p value* and *Hazard Ratios* when p-value < 0.05) of classical prognostic factors derived from initial staging and of metrics derived from initial FDG-PET, on univariate analysis.**

Significant p-value in bold

For EWS, anatomic primary site corresponds to primary tumor site (axial/pelvic vs peripheral).

For OST, histologic subtypes compares Osteoblastic vs Chondroblastic/Telangiectatic subtypes.

(EWS: Ewing sarcoma; OST: Osteosarcoma; SUV: Standard Uptake Value; TLG: Total Lesion Glycolysis MTV: Metabolic tumor volume; HGRE: High Grey Level Run Emphasis; SZHGE: Short-Zone High Gray-level Emphasis; ZNLU: Zone Length Non Uniformity)
