# Supplementary material for: Prognostic value of FDG-PET indices for the assessment of histological response to neoadjuvant chemotherapy and outcome in pediatric patients with Ewing sarcoma and osteosarcoma
Source: PLoS One. 2017 Aug 25;12(8):e0183841. doi: 10.1371/journal.pone.0183841 (PMC5571925; doi:10.1371/journal.pone.0183841)
Supplement: S2 Table — No Hazard Ratio is shown as no significant p-value was observed.(EWS: Ewing sarcoma; OST: Osteosarcoma; CI: conventional imaging; SUV: Standard Uptake Value; TLG: Total lesion glycolysis; MTV: Metabolic tumor volume). (DOC) [file pone.0183841.s002.doc]

|  | **EWS** | | **OST** | |
| --- | --- | --- | --- | --- |
| Parameter | PFS | OS | PFS | OS |
| PERCIST | 0.645 | 0.060 | 0.460 | 0.439 |
| CI response | 0.62 | 0.814 | 0.349 | 0.359 |
| SUVmax_2_ | 0.248 | 0.563 | 0.291 | 0.39 |
| ΔSUVmax | 0.389 | 0.33 | 0.98 | 0.58 |
| SUVpeak_2_ | 0.106 | 0.924 | 0.359 | 0.436 |
| ΔSUVpeak | 0.415 | 0.806 | 0.948 | 0.484 |
| SUVmean_2_ | 0.307 | 0.962 | 0.296 | 0.33 |
| ΔSUVmean | 0.38 | 0.411 | 0.968 | 0.489 |
| TLG_2_ | 0.642 | 0.449 | 0.722 | 0.781 |
| ΔTLG | 0.379 | 0.336 | 0.592 | 0.432 |
| MTV_2_ | 0.992 | 0.377 | 0.967 | 0.459 |
| ΔMTV | 0.5 | 0.353 | 0.618 | 0.855 |

**S2 Table :** **Prognostic values (*p value*) of indices derived from post-CHT FDG-PET on univariate analysis.**

No Hazard Ratio is shown as no significant p-value was observed.

(EWS: Ewing sarcoma; OST: Osteosarcoma; CI: conventional imaging; SUV: Standard Uptake Value; TLG: Total lesion glycolysis; MTV: Metabolic tumor volume)
