# Supplementary material for: Prognostic value of FDG-PET indices for the assessment of histological response to neoadjuvant chemotherapy and outcome in pediatric patients with Ewing sarcoma and osteosarcoma
Source: PLoS One. 2017 Aug 25;12(8):e0183841. doi: 10.1371/journal.pone.0183841 (PMC5571925; doi:10.1371/journal.pone.0183841)
Supplement: S3 Table — (EWS: Ewing sarcoma; OST: Osteosarcoma; SUV: Standard Uptake Value; TLG: Total Lesion Glycolysis MTV: Metabolic tumor volume). (DOC) [file pone.0183841.s003.doc]

|  | **EWS** | | **OST** | |
| --- | --- | --- | --- | --- |
| Parameter | Spearman's rho | p | Spearman's rho | p |
| ΔSUVmax | 0.469 | 0.066 | 0.176 | 0.41 |
| ΔSUVpeak | 0.392 | 0.133 | 0.137 | 0.522 |
| ΔSUVmean | 0.631 | 0.122 | 0.228 | 0.284 |
| ΔTLG | 0.111 | 0.692 | 0.062 | 0.773 |
| ΔMTV | -0.038 | 0.892 | 0.028 | 0.897 |

**S3 Table**: **Correlations (Spearman coefficients) between delta and histological regression**

(EWS: Ewing sarcoma; OST: Osteosarcoma; SUV: Standard Uptake Value; TLG: Total Lesion Glycolysis MTV: Metabolic tumor volume)
